# Use of monitoring indicators in hospital management of antimicrobials

**DOI:** 10.1186/s12879-021-06542-5

**Published:** 2021-08-17

**Authors:** Ravena Melo Ribeiro da Silva, Simonize Cunha B. de Mendonça, Ingrid Novaes Leão, Quesia Nery dos Santos, Alessandra Macedo Batista, Matheus Santos Melo, Milena da Motta Xavier, Lucindo José Quintans Júnior, Wellington Barros da Silva, Iza Maria Fraga Lobo

**Affiliations:** 1 Department of Pharmacy, Federal University of Sergipe, São Cristóvão, Se Brazil; 2 Department of Nursing, Federal University of Sergipe, São Cristóvão, Se Brazil; 3 Department of Physiology, Federal University of Sergipe, São Cristóvão, Se Brazil; 4 University Hospital, Federal University of Sergipe, São Cristóvão, Se Brazil; 5 General Hospital Prado Valadares, Jequié, Ba Brazil; 6 Emergency Hospital of Sergipe, Aracaju, Se Brazil

**Keywords:** Anti-infective agents, Antimicrobial stewardship, Drug resistance

## Abstract

**Background:**

According to the literature, 25% to 50% of antimicrobials prescribed in hospitals are unnecessary or inappropriate, directly impacting antimicrobial resistance. Thus, the present study aimed to evaluate the use of antimicrobials in a university hospital in Northeast Brazil, using days of therapy (DOT) and length of therapy (LOT) indicators in accordance with the latest national and international recommendations for monitoring the use of antimicrobials.

**Methods:**

This is an observational, prospective analytical study conducted in a teaching hospital, with 94 active beds, distributed between the intensive care unit (ICU), the surgical clinic (SUR), the medical clinic (MED), the pneumology/infectology department (PNE/INF) and pediatrics (PED). The duration of the study was from the beginning of January to the end of December 2018.

**Results:**

During the study period, a total of 11,634 patient-days were followed up and 50.4% of the patients were found to have received some antimicrobial, with a significant reduction in use of 1% per month throughout the year. Patients were receiving antimicrobial therapy for 376 days in every 1000 days of hospitalization (LOT = 376/1000pd). Overall, the 1st-generation cephalosporins and fluoroquinolones were the most used in respect of the number of prescriptions and the duration of therapy. The calculated global DOT/LOT ratio showed that each patient received an average of 1.5 antimicrobials during the hospital stay. The incidence of antimicrobial resistance, globally, for both methicillin-resistant *Staphylococcus aureus* (methicillin R), Carbapenem-resistant *Klebsiella pneumoniae*, *Pseudomonas aeruginosa* and *Acinetobacter baumannii* (Carbapenem R), was 1 per 1000 patient-days.

**Conclusions:**

The results obtained from the analyses revealed that half of the patients admitted to the hospital who took part in the study were exposed to the use of antimicrobials at some point during their stay. Although moderate, it is noteworthy that there was a decline in the use of antimicrobials throughout the year. The indicators used in this study were found to be very effective for gathering data on the use of antimicrobials, and assessing the results of the initiatives taken as part of the Stewardship program.

**Supplementary Information:**

The online version contains supplementary material available at 10.1186/s12879-021-06542-5.

## Background

Microbial resistance is one of the main global public health problems, and although a natural biological phenomenon, it is occurring at an accelerated rate due to the excessive use of antimicrobials (ATMs). According to the literature, 25% to 50% of antimicrobial prescriptions in hospitals are unnecessary or inappropriate, and result in an increased length of hospital stays, morbidity, health care costs, and antimicrobial resistance [1–6].

Diagnostic uncertainty, the incorrect choice of medications, improper treatment duration, the presence of complex comorbidities, the incorrect interpretation of microbial results, and a lack of surveillance to monitor the use of antimicrobials are the main factors that are responsible for the inappropriate use of these drugs [5, 7, 8].

In response to this growing problem, the Centers for Disease Control and the World Health Organization, together with the United Nations member countries, including Brazil, have been encouraging the implementation of antimicrobial stewardship programs (ASPs), which are a set of interventions, audits, and feedback, to optimize the use of ATMs [2, 3, 9, 10]. In July 2019, Brazilian Health Regulatory Agency (ANVISA) set up the Brazil Stewardship Project [6] to assess the national panorama of antimicrobial use management programs, and to encourage the implementation by the country's health service providers of strategies to prevent the spread of resistant microorganisms in Brazil.

Several measures have been used to evaluate ATM use and consumption. The defined daily dose (DDD), which expresses the daily dose, in grams, of a drug used is the oldest and most used, but this measure is not recommended for pediatric populations, as individual doses vary according to the child's weight. Thus, alternative metrics were proposed and adopted, such as the days of therapy (DOT) measure, equivalent to the number of days a patient receives an antimicrobial agent, regardless of the dose, with any dose received during a 24-h period representing 1 DOT; and the length of therapy (LOT) measure, referring to the number of days the patient is on antimicrobial therapy, regardless of the number of drugs, meaning that LOT will always be lower or equal to DOT. The higher the DOT and LOT, the longer the patient is exposed to the use of antimicrobials. The DOT/LOT ratio can be useful to assess the number of antimicrobials prescribed per patient, with a ratio of 1 representing monotherapy, and a ratio of > 1 representing combination therapy [1, 3, 9, 11, 12].

In this study, measures and indicators of antimicrobial use were particularly important, as we used DOT and LOT values and the DOT/LOT ratio to explore and monitor antimicrobial use. Data on the use of antimicrobials in Brazil at the patient level is limited, and, to date, this is the first study in the country to use DOT and LOT indicators across several hospital wards. Thus, the aim of the study was to evaluate the use of antimicrobials in a university hospital in Northeast Brazil, using these indicators in accordance with the latest national and international recommendations [3, 6].

## Methods

### Design and setting

This is an observational, prospective analytical study conducted between 1 January and 31 December 2018 in a teaching hospital with 94 active beds, distributed between the intensive care unit (ICU), the surgical clinic (SUR), the medical clinic (MED), the pneumology/infectology department (PNE/INF) and pediatrics (PED).

From the 2nd quarter of the study, the hospital started implementing actions as part of a new stewardship program, which continued throughout the year. The program involved professionals from the Health Care-Related Infection Control Service, the Care Risk Management Unit, the hospital pharmacy, microbiology, infectology, intensive care, pediatrics, and other specialty units, in a joint effort to implement and monitor measures to improve the use of antimicrobials.

### Inclusion and exclusion criteria

Data analysis included the prescriptions of a sample of adult and pediatric patients admitted to the wards (including those who did not receive antimicrobials). The exclusion criteria were patients with a stay of fewer than 24 h. Preoperative antimicrobial prophylaxis given in the operating theatre under the standard protocol does not appear in the ordinary prescriptions and, thus, was excluded from the analysis.

### Data source

The sample size was obtained through the sample calculation for a finite population, considering the population admitted to the unit in 2017. A confidence level of 95% and 5% error was established. Next, stratified sampling was carried out with proportional distribution among the units, that is, the sample size obtained was divided into the proportions of the population of individuals admitted to each unit.

The review of prescriptions and data extraction was performed by a researcher using an electronic database created especially for this purpose. The data about the use of antimicrobials were obtained from the records of individual prescriptions for antimicrobials maintained by the hospital pharmacy.

### Outcome measures

Primary outcomes were represented by the distribution of antimicrobial use, individually or by class, and employed time series analyses, expressed as DOT and LOT per 1000 patient-days (pd), and the DOT/LOT ratio. When only one antimicrobial of a class was used in the hospital, they were identified by the name of the substance rather than the class name.

Secondary outcomes described the percentage of patients using antimicrobials, the number of antimicrobial prescriptions; the incidence of methicillin-resistant *Staphylococcus aureus* (methicillin R), Carbapenem-resistant *Klebsiella pneumoniae*, *Pseudomonas aeruginosa* and *Acinetobacter baumannii* (Carbapenem R) per 1000 patient-days (pd); and the diagnoses performed by infectious disease specialists, through clinical practice and/or guided by culture, for the use of antimicrobials.

Data about antimicrobial resistance were obtained through active search forms, surveillance of positive cultures, and data gathered by the health care-related infection control service of the hospital as part of their work to prevent microbial resistance. The incidence densities of antimicrobial resistance were obtained through the ratio between the number of patients infected by the resistant pathogen divided by the number of patient-days, multiplied by 1000.

### Statistical analysis

Data entry and storage were performed using Microsoft Excel 2019 (Microsoft Corporation, Redmond, WA, USA), and Stata software version 15.1 (StataCorp, College Station, Texas, USA) was used for the statistical analysis. The variables were expressed as means and percentages. The normality of data distribution was verified and compared using the ANOVA test or the Kruskal–Wallis test with respective post-tests, as appropriate.

For the analysis of time trend, generalized linear regression was used with the Prais-Winsten model, which allows the correction of first-order serial autocorrelation, indicated for time series analysis. The level of statistical significance was set at 0.05.

## Results

During the study period, a total of 11,634 patient-days were followed up and an average of 50.4% of patients was exposed to some antimicrobial (Additional file 1). The intensive care unit was the ward that contained the largest proportion of patients using ATM (86%; ICU vs SUR, MED; p = 0.000), followed by pediatrics (64%; PED vs SUR; p = 0.002) and the pneumology/infectology department (62%; PNE/INF vs SUR; p = 0.005) (Table 1).

**Table 1:** Distribution of antimicrobial use, individually or by class, expressed in percentages, DOT and LOT per 1000 patient-days (pd), and the DOT/LOT ratio, by hospital ward, 2018

Variables	Wards					
	SUR	MED	PNE/INF	PED	ICU	p-value
Use of antimicrobials (%)	38.0^a^	42.0^b^	62.0^c(a)^	64.0^d(a)^	86.0^e(ab)^	0.000*
Number of prescriptions (%)						
First-generation cephalosporins IV/PO	49.4^a(bce)^	3.9^b^	1.4^c^	34.5^d(bc)^	9.6^e^	0.000*
Fluoroquinolones IV/PO	20.5^a^	14.2^b^	11.2^c^	3.6^d(abe)^	13.7^e^	0.000*
Carbapenems IV	1.7^a^	11.6^b(a)^	8.8^c^	6.3^d^	19.6^e(ad)^	0.000*
Third-generation cephalosporins IV	6.5^a^	14.8^b(ae)^	8.5^c^	9.2^d^	4.1^e^	0.011*
Metronidazole IV/PO	10.1^a^	5.8^b^	1.7^c(ae)^	6.3^d^	11.3^e^	0.001*
Cefepime IV	0.8^a^	10.3^b(a)^	15.6^c(a)^	6.0^d^	7.2^e^	0.000*
Clindamycin IV	9.1	7.1	5.1	2.1	7.6	0.102
Antifungals IV/PO	0.2^a^	7.1^b(a)^	16.0^c(ad)^	4.5^d^	6.9^e^	0.000*
Glycopeptides IV	1.1^a^	6.5^b^	3.4^c^	3.0^d^	10.7^e(acd)^	0.001*
Azithromycin IV/PO	0^a^	2.6^b^	9.5^c(abe)^	6.3^d(a)^	1.7^e^	0.000*
Penicillins^1^ IV/PO	0.2^a^	10.3^b(ae)^	3.4^c^	8.3^d(ae)^	1.0^e^	0.000*
Co-trimoxazole IV/PO	0^a^	3.2^b^	10.9^c(abe)^	3.6^d(a)^	3.1^e^	0.000*
Aminoglycosides IV	0.4^a^	2.6^b^	4.4^c^	6.5^d(a)^	3.4^e^	0.017*
ID LOT/1000pd	300^a^	262^b^	470^c(ab)^	382^d(b)^	753^e(abcd)^	0.000*
ID DOT/1000pd						
Fluoroquinolones IV/PO	108^a^	57^b^	71^c^	28^d(ae)^	147^e^	0.001*
First-generation cephalosporins IV/PO	123^a(bc)^	6^b^	6^c^	94^d(bc)^	73^e(bc)^	0.000*
Carbapenems IV	11^a^	43^b^	84^c(a)^	40^d^	368^e(abd)^	0.000*
Third-generation cephalosporins IV	43	57	64	61	35	0.519
Cefepime IV	8^a(ce)^	32^b^	95^c^	47^d^	114^e^	0.000*
Metronidazole IV/PO	56^a^	12^b(e)^	8^c(ade)^	59^d^	119^e^	0.000*
Antifungals IV/PO	1^a(ce)^	24^b^	83^c^	40^d^	118^e^	0.000*
Co-trimoxazole IV/PO	0^a^	4^b(c)^	121^c(ab)^	25^d^	72^e(a)^	0.000*
Clindamycin IV	47	28	37	12	73	0.099
Glycopeptides IV	8^a^	25^b^	36^c^	10^d^	183^e(abcd)^	0.000*
Aminoglycosides IV	6	16	32	34	83	0.055
Penicillins^1^ IV/PO	1^a^	35^b(a)^	18^c^	38^d(ae)^	13^e^	0.000*
Azithromycin IV/PO	0^a(cd)^	10^b(c)^	40^c^	31^d^	23^e^	0.000*
ID DOT/LOT ratio	1.4^a^	1.3^b^	1.5^c^	1.4^d^	2.0^e(abcd)^	0.000*

*ID* Incidence Density, *SUR* Surgical Clinic, *MED* Medical Clinic, *PNE/INF* Pneumology/Infectology, *PED *Pediatrics, *ICU* Intensive Care Unit

^1^Penicillin/penicillin with beta-lactamase inhibitor

^a,b,c,d,e^Statistical differences per line (Bonferroni *p < 0.05)

Overall, the most prescribed classes were the 1^st^-generation cephalosporins (25.0%), fluoroquinolone (13.2%), and carbapenems (8.4%) (Additional file 1). The 1st-generation cephalosporins were the most prescribed classes, both in the surgical clinic (49.4%; SUR vs MED, PNE/INF, ICU; p = 0.000) and in pediatrics (34.5%; PED vs MED, PNE/INF; p = 0.000). The medical clinic had a higher number of prescriptions of 3rd generation cephalosporins (14.8%; MED vs SUR, p = 0.036; MED vs ICU, p = 0.002), the pneumology/infectology department had a higher number of antifungals (16.0%; PNE/INF vs SUR, PED; p = 0.000) and the ICU had the most prescriptions of carbapenems (19.6%; ICU vs SUR, PED; p = 0.000) (Table 1).

Patients were receiving antimicrobial therapy for 376 of every 1000 days of hospitalization (LOT = 376/1000pd) (Additional file 1). Comparing hospital wards, patients were exposed to ATMs for the longest time in the ICU (753 LOT/1000pd; ICU vs SUR, MED, PNE/INF, PED; p = 0.000), followed by the pneumology/infectology department (470 LOT/1000pd; PNE/INF vs SUR, MED; p = 0.000). The calculated global DOT/LOT ratio showed that each patient received an average of 1.5 antimicrobials during the hospital stay (Additional file 1), with a higher number within the ICU (2.0 DOT/LOT; ICU vs SUR, MED, PNE/INF, PED; p =  < 0.000) (Table 1).

In the hospital, the therapy time was longer for fluoroquinolone (75 DOT/1000pd), followed by 1st-generation cephalosporins (66 DOT/1000pd), and carbapenems (61 DOT/1000pd) (Additional file 1). Exposure time to 1st-generation cephalosporins in the surgical clinic (123 DOT/1000pd; SUR vs MED, PNE/INF; p = 0.000) and in pediatrics (94 DOT/1000pd; PED vs MED, PNE/INF; p = 0.000) was superior to the other antimicrobial classes. Higher exposures were also observed for fluoroquinolone, and 3rd generation cephalosporins in the medical clinic (57 DOT/1000pd, each), co-trimoxazole in pneumology/infectology department (121 DOT/1000pd; PNE/INF vs SUR, MED; p = 0.001) and carbapenems in the ICU (368 DOT/1000pd; ICU vs SUR, MED, PED; p = 0.000) (Table 1).

The incidence of antimicrobial resistance globally, for both Methicillin ^R^ and Carbapenem ^R^, was 1 per 1000 patient-days, each (Additional file 2), presenting a higher rate of Carbapenem ^R^ in the ICU (ID = 6/1000pd; ICU vs SUR, PNE/INF, PED; p = 0.023).

Overall, diagnoses for the use of antimicrobials involved surgical prophylaxis (35.7%), respiratory infections (24.1%), and sepsis (14.3%) (Additional file 3). Surgical prophylaxis was the main indication for the use of antimicrobials in the surgical clinic (80.8%; SUR vs MED, PNE/INF, p = 0.000; SUR vs ICU; p = 0.014), and in the ICU. Respiratory infections were higher in the medical clinic (22.8%) and pneumology/infectology department (39.1%; PNE/INF vs SUR; p = 0.008). The medical clinic also showed a significant difference in the number of abdominal infections compared to pediatrics (19.3%; MED vs PED p = 0.047). In pediatrics, there was a prevalence of indication of the use of antimicrobials for surgical prophylaxis (35.8%; PED vs MED, PNE/INF; p = 0.027) and respiratory infections (35.8%; PED vs SUR; p = 0.005) (Table 2).

**Table 2:** Distribution of diagnoses related to the use of antimicrobials, by hospital ward, 2018

Diagnostic (%)	Wards					
	SUR	MED	PNE/INF	PED	ICU	p-value
Surgical prophylactic	80.8^a(bce)^	0^b^	0^c^	35.8^d(bc)^	34.7^e^	0.000*
Respiratory infection	4.2^a(cd)^	22.8^b^	39.1^c^	35.8^d^	20.8^e^	0.010*
Sepsis	0^a^	19.3^b^	19.6^c^	6.7^d^	32.7^e(a)^	0.023*
Skin/soft tissue infection	2.5	17.5	16.3	6.7	1.0	0.074
Urinary infection	2.5	17.5	14.1	4.2	4.0	0.957
Abdominal infection	7.5^a^	19.3^b(d)^	1.1^c^	0.8^d^	6.9^e^	0.047*
Others	2.5	3.5	9.8	10.0	0	0.154

*SUR* Surgical Clinic, *MED* Medical Clinic, *PNE/INF* Pneumology/Infectology, *PED* Pediatrics, *ICU* Intensive Care Unit.

Others—Surgical Site Infection, Visceral Leishmaniasis, Febrile Neutropenia, Bacterial Endocarditis.

^a,b,c,d,e^Statistical differences per line (Bonferroni *p < 0.05)

### Trend

The time series analyses revealed a significant reduction in the use of antimicrobials, globally (− 1.0%; p = 0.026), and, in the surgical clinic (− 1.6%; p = 0.005); however, there was an increase in the medical clinic (2.6%; p = 0.021) (Additional files 1, 4). Overall, there was a significant decrease in the use of aminoglycosides (− 0.3%; p = 0.041), and a significant increase in carbapenems (0.4%; p = 0.018). The surgical clinic obtained a significant reduction in the number of aminoglycosides prescriptions (− 0.1%; p = 0.037). In the medical clinic, there was a reduction in the number of penicillins (− 1.9%; p = 0.001) and an increase in the number of carbapenems (1.0%; p = 0.002). In the pneumology/infectology department, reductions were observed for 1st-generation cephalosporins (− 0.3%; p = 0.008) and aminoglycosides (− 1.1%; p = 0.017) and an increase in the number of metronidazole (0.9%; p = 0.027). In pediatrics, there was an increase in the number of azithromycin prescriptions (0.9%; p = 0.004). There was no significant change in the ICU prescriptions (Additional files 1, 4).

There were no significant changes in the duration of antimicrobial therapy (LOT/1000pd) and the DOT/LOT ratio, in general, and between the units analysed (Additional files 1, 4). Regarding the time of use of single antimicrobials, overall significant decreases were observed in the use of 1st-generation cephalosporins (− 2.9 DOT/1000pd; p = 0.005), and aminoglycosides (− 3.5 DOT/1000pd; p = 0.009). These falls were mostly observed in the surgical clinic (1st-generation cephalosporins − 10.6 DOT/1000pd, p = 0.000; and aminoglycosides − 1.8 DOT/1000pd; p = 0.037), but there was an increased time of use of glycopeptides (1.7 DOT/1000pd; p = 0.046). In the medical clinic, there was a reduced time of use of penicillins (− 4.9 DOT/1000pd; p = 0.034) but an increase in the time of use of carbapenems (5.6 DOT/1000pd; p = 0.009). In the pneumology/infectology department, the only significant change observed was a reduction in the time of use of aminoglycosides (− 7.3 DOT/1000pd; p = 0.008). In pediatrics, the time of use of azithromycin increased (3.6 DOT/1000pd; p = 0.021). In the ICU there was a decrease in the time of use of clindamycin (− 23.5 DOT/1000pd; p = 0.015), and an increase for carbapenems (24.4 DOT/1000pd; p = 0.044).

Overall, incidence rates of antimicrobial resistance revealed an increase in respect of Methicillin ^R^ (0.1; p = 0.031), especially in the surgical clinic (0.1; p = 0.002) and in the ICU (1.0; p = 0.024) (Additional files 2, 4).

## Discussion

The present study found that 50.4% of patients received some antimicrobial. A large study carried out by Versporten et al. [7], reported that in Latin American hospitals ATMs were used in an average of 36.8% patients, ranging from 32.5 to 43.4%. However, the literature describes a broader range that extends from 22 to 76% of hospitalized patients receiving at least one antimicrobial during their hospital stay [1, 4, 8, 9, 12]. Even though the results presented in this study are within this range, these studies demonstrate variations that may be related to the type of care provided in each hospital, where the need for antimicrobial indication may be higher or lower. Furthermore, diagnostic uncertainty, the incorrect choice of medications, improper treatment duration, the presence of complex comorbidities, the incorrect interpretation of microbial results, and a lack of surveillance to monitor the use of antimicrobials are responsible for the inappropriate use of these drugs [5, 7, 8].

The greatest number of patients exposed to the use of antimicrobials was in the intensive care unit (86%), followed by pediatrics (64%) and the pneumology/infectology department (62%). The predominance of the use of ATMs in the ICU was also observed in a study of prevalence on health-related infections in Austria, in which 67.9% of patients in this ward were exposed to some antimicrobial, with other studies indicating variations from 55.1% to 57% [1, 4, 7]. The use of antimicrobials is greater in the ICU compared to other wards since the prevalence of infections in ICUs is higher, this is because these units tend to care for critically ill patients, with serious immunological dysfunctions and compromised natural barriers [1]. In addition, in our study, time trend analyses showed a reduction in the percentage of antimicrobial use in the surgical clinic and an increase in the medical clinic. The reduction in antimicrobial prescriptions in the postoperative period reflects changes in the conduct of prescribers regarding the implementation of the stewardship program, which in its guidance points out that data from the literature suggests that the post-operative continuation of antibiotic prophylaxis produces no additional benefit in reducing the incidence of surgical site infection [13].

The LOT indicator showed that patients were receiving antimicrobial therapy for 376 days for every 1000 days of hospitalization (LOT = 376/1000pd). The LOT indicator is rarely found in the literature to express the use of antimicrobials; however, a study in the United States found an average LOT of 536 (median, 529; range, 427–684) per 1000 patient-days in 70 American hospitals [12], a value above that observed in this study. The reduced time of exposure to the use of antimicrobials in the hospital analysed demonstrates the role of the multidisciplinary team to contain the indiscriminate use of these medications. In low and middle-income countries (LMICs), studies on different monitoring methods for antimicrobial management are scarce. A recent meta-analysis of 221 studies using clinical trials, interrupted time series, and other methods evaluated the effectiveness of antibiotic management programs, but few of the studies represented LMICs [14].

As mentioned above, the ICU and the pneumology/infectology department presented LOTs well above the overall hospital rate, reflecting the severity and frequency of infections in patients assisted in these two wards, which require more frequent, intense, and longer antimicrobial therapy [1]. The calculated global DOT/LOT ratio showed that each patient received an average of 1.5 antimicrobials during their hospital stay, with a higher number prescribed within the ICU (2.0 DOT/LOT). The literature points to similar values ranging from 1.3 to 2.1 antimicrobials per patient [4, 5, 8]. Combination therapy, in specific circumstances, can be useful to expand antimicrobial coverage and thus reduce the risk of the pathogen being insusceptible to the ATM, such as in cases of: severe community-acquired pneumonia; severe hospital-acquired or ventilator-associated pneumonia or when there is a high risk of resistance in hospital-acquired or ventilator-associated pneumonia; multi-drug or extensively drug-resistant gram-negative infections; and severe group A streptococcal infections. However, in situations other than these, this approach can end up being harmful to the patient and the community [15].

Overall, 1^st^-generation cephalosporins stood out, both in the number of prescriptions and in the duration of therapy, especially in the surgical, and pediatric wards; however, the data reveal that there is a tendency to reduce the use of these drugs. The use of 1^st^-generation cephalosporins in the hospital under study is related to the surgical profile of the institution, with the use of old protocols. The hospital performs, on average, 400 elective surgeries per month, mainly abdominal and intra-abdominal, gastrointestinal tract, reproductive system, and breast surgeries. 1^st^-generation cephalosporins are the first choice for most surgeries, being associated with the use of metronidazole in gastrointestinal procedures, with the possibility extension of their use to 24 or 48 h.

Another class that stands out is the quinolones, especially in surgery. There must be a reassessment regarding their use, as in early January 2019, the European Medicines Agency [16] released a report recommending the suspension and restriction of the use of some antibiotics, among them quinolones and fluoroquinolones, because of the serious, disabling, and potentially permanent side effects that these drugs have presented. The literature also reports a significant correlation between the use of cephalosporins and fluoroquinolones with the increase in carbapenem-resistant *enterobacteriaceae* [17].

The medical clinic had a higher number of prescriptions of 3rd generation cephalosporins, with longer exposure to quinolones. The data showed some reduction in these variables over the year, but without a statistically significant change. On the other hand, a significant increase was observed in both the number of prescriptions and the time of exposure to carbapenems. In the pneumology/infectology department the use of antifungals and longer use of co-trimoxazole predominated, with no significant increases. Despite this, there was a significant reduction in the use of aminoglycosides. In the ICU, carbapenems stood out both in respect of the number of prescriptions and the duration of the therapy, with a significant increase in this exposure ​​throughout the study. The high use of carbapenems in the ICU may explain the high incidence of Carbapenem ^R^. There was also an increase in the use of Methicillin ^R^ in the ICU and the surgical clinic. At present, there is no expectation of the availability of new antibiotics to replace carbapenems. The implementation and adherence to simple infection control measures such as hand hygiene, the correct cleaning of the environment, and contact precautions still work as basic strategies to prevent the transmission of multi-resistant bacteria [17].

A study conducted by Kimura [18], which evaluated the long-term effects of antibiotic administration programs at a university hospital in Japan, found that therapy days per 1000 patient-days were higher for 1^st^-generation cephalosporins (45 DOT/1000pd). The time of use of quinolones (4.7 DOT/1000pd) and carbapenems (13.5 DOT/1000pd) were well below the values verified in this study. However, studies show results ranging from 25.8 DOT/1000pd to 132.3 DOT/1000pd for quinolones and 8.7 DOT/1000pd to 39.1 DOT/1000pd for carbapenems [2, 9, 10], this variation may be related to the lack of uniformity in data collection, which highlights the importance of standardizing the methods of obtaining these rates to allow reliable comparability of the data.

Surgical prophylaxis, respiratory infections, and sepsis were the most described diagnoses for the use of antimicrobials. Surgical prophylaxis was the main diagnostic for the use of antimicrobials in the surgical clinic, which is in line with the greater number of indications of 1^st^-generation cephalosporins in this ward. A similar prophylactic use of antimicrobials was also reported by Talaam et al. [5] in a rural hospital in western Kenya, where it was responsible for 56.3% of antimicrobial indications. Gutema et al. [8] reported that the prophylactic use of antimicrobials represented 41.3% of the prescriptions in a surgical clinic and 8.5% in a medical clinic. Respiratory infections prevailed in respect of the medical clinic, and pneumology/infectology department. The pediatric ward had a higher prevalence of indication of antimicrobial therapy in both surgical prophylaxis and respiratory infections. This reflects the characteristics of the wards described and corroborates other studies that indicate that approximately 50% of antimicrobials are used in the treatment of respiratory conditions [4, 8, 9]. In the ICU, the main diagnostic indications were for surgical prophylaxis, explained by the fact that it is a transition ward between the operating room, welcoming the patient in the immediate postoperative period, and the surgical ward. Another important indication refers to the treatment of sepsis. Sepsis is a serious public health problem in ICUs in Brazil and is the second leading cause of mortality within this environment. Empirical treatment with antibiotics is usually initiated with broad-spectrum drugs, such as carbapenems, as observed in this study, possibly due to the number of multidrug-resistant strains isolated in these patients [19].

The strengths of the current study are its prospective design with the use of time series analyses, the direct investigation of the prescriptions that allows a more detailed analysis of the use of antimicrobials, and the use of DOT and LOT indicators for monitoring the use of antimicrobials. However, the study has some limitations: First, it was conducted in a single center; Second, comorbidities were not evaluated; and third, the appropriateness of the prescriptions was not addressed.

## Conclusion

The results obtained from the analyses revealed that half of the patients admitted to the hospital under study were exposed to the use of antimicrobials at some point during their stay. The ICU was the unit with the greatest number and time of exposure to antimicrobials, with prophylaxis being the main diagnosis for its use, with emphasis on 1st generation cephalosporins and quinolones. Although moderate, it is noteworthy that there was a decline in the use of antimicrobials throughout the year.

The DOT, and LOT per 1000pd indicators used in this study were found to be very effective for obtaining data regarding the use of antimicrobials. They also provided feedback to health professionals in respect of the measures undertaken as part of the Stewardship program implemented in the hospital to improve patient care and safety. It is hoped that this study will encourage other hospitals to use these measures to monitor the use of antimicrobials, allowing comparison of data on a national and international basis.

## Supplementary Information

**Additional file 1.** Global distribution of antimicrobial use, individually or by class, and employed time series analyses, expressed in percentages, DOT and LOT per 1000 patient-days (pd), and the DOT/LOT ratio, 2018.

**Additional file 2.** Global incidence of antimicrobial resistance per 1000 patient-days, 2018.

**Additional file 3.** Global distribution of diagnoses related to the use of antimicrobials, 2018.

**Additional file 4.** Temporal trends in the use of antimicrobials, by hospital ward, 2018.

## Data Availability

The datasets used and/or analysed during the current study are available from the corresponding author on reasonable request.

## Abbreviations

ATM: Antimicrobials
Carbapenem ^R^: Carbapenem Resistance in *Klebsiella pneumoniae*, *Pseudomonas aeruginosa*, and *Acinetobacter baumannii*
DDD: Defined Daily Dose
DOT: Days of Therapy
ICU: Intensive Care Unit
LMICs: Low and middle-income countries
LOT: Length of therapy
MED: Medical clinic
Methicillin ^R^: Methicillin-resistant Staphylococcus aureus
PED: Pediatrics
PD: Patient-days
PNE/INF: Pneumology/infectology
SUR: Surgical clinic
