# Supplementary material for: Use of monitoring indicators in hospital management of antimicrobials
Source: BMC Infect Dis. 2021 Aug 17;21:827. doi: 10.1186/s12879-021-06542-5 (PMC8369325; doi:10.1186/s12879-021-06542-5)
Supplement: Supplementary file 1 — Additional file 1. Global distribution of antimicrobial use, individually or by class, and employed time series analyses, expressed in percentages, DOT and LOT per 1000 patient-days (pd), and the DOT/LOT ratio, 2018. [file 12879_2021_6542_MOESM1_ESM.docx]

**Additional file 1.** Global distribution of antimicrobial use, individually or by class, and employed time series analyses, expressed in percentages, DOT and LOT per 1000 patient-days (pd), and the DOT/LOT ratio, 2018.

| **VARIABLES** | **Value** | **Monthly Variation** | **p-value** |
| --- | --- | --- | --- |
| **Use of antimicrobials (%)** | 50.4 | -1.0 | 0.026* |
| **Number of prescriptions (%)** |  |  |  |
| First-generation cephalosporins IV/PO | 25 | -0.6 | 0.295 |
| Fluoroquinolones IV/PO | 13.2 | -0.0 | 0.909 |
| Carbapenems IV | 8.4 | 0.4 | 0.018* |
| Third-generation cephalosporins IV | 7.9 | -0.0 | 0.909 |
| Metronidazole IV/PO | 7.5 | 0.2 | 0.572 |
| Cefepime IV | 6.9 | 0.1 | 0.556 |
| Clindamycin IV | 6.3 | -0.5 | 0.303 |
| Antifungals IV/PO | 6.1 | 0.2 | 0.235 |
| Glycopeptides IV | 4.3 | 0.2 | 0.450 |
| Azithromycin IV/PO | 3.7 | 0.2 | 0.276 |
| Penicillins^1^ IV/PO | 3.7 | 0.0 | 0.656 |
| Co-trimoxazole IV/PO | 3.7 | 0.1 | 0.257 |
| Aminoglycosides IV | 3.3 | -0.3 | 0.041* |
| **ID LOT/1000pd** | 376 | -0.7 | 0.874 |
| **ID DOT/1000pd** |  |  |  |
| Fluoroquinolones IV/PO | 75 | -1.6 | 0.382 |
| First-generation cephalosporins IV/PO | 66 | -2.9 | 0.005* |
| Carbapenems IV | 61 | 2.4 | 0.194 |
| Third-generation cephalosporins IV | 53 | 0.7 | 0.562 |
| Cefepime IV | 46 | -0.3 | 0.878 |
| Metronidazole IV/PO | 42 | 1.1 | 0.659 |
| Antifungals IV/PO | 39 | 1.4 | 0.406 |
| Co-trimoxazole IV/PO | 38 | 3.0 | 0.246 |
| Clindamycin IV | 35 | -2.7 | 0.366 |
| Glycopeptides IV | 28 | 1.3 | 0.355 |
| Aminoglycosides IV | 24 | -3.5 | 0.009* |
| Penicillins^1^ IV/PO | 20 | -0.4 | 0.353 |
| Azithromycin IV/PO | 19 | 0.9 | 0.488 |
| **ID DOT/LOT ratio** | 1.5 | -0.0 | 0.812 |

**Legend:** ID – Incidence Density, ^1^ penicillin/penicillin with beta-lactamase inhibitor. *Prais-Winstein Regression (p < 0.05).
