# Supplementary material for: Use of monitoring indicators in hospital management of antimicrobials
Source: BMC Infect Dis. 2021 Aug 17;21:827. doi: 10.1186/s12879-021-06542-5 (PMC8369325; doi:10.1186/s12879-021-06542-5)
Supplement: Supplementary file 2 — Additional file 2. Global incidence of antimicrobial resistance per 1000 patient-days, 2018. [file 12879_2021_6542_MOESM2_ESM.docx]

**Additional file 2.** Global incidence of antimicrobial resistance per 1000 patient-days, 2018.

| **ANTIMICROBIAL RESISTANCE** | **Incidence** | **Monthly Variation** | **p-value** |
| --- | --- | --- | --- |
| Methicillin ^R^ | 1 | 0.1 | 0.031* |
| Carbapenem ^R^ | 1 | -0.0 | 0.152 |

**Legend:** Methicillin R - Methicillin-resistant *Staphylococcus aureus*; Carbapenem R - Carbapenem Resistance in *Klebsiella pneumoniae, Pseudomonas aeruginosa*, and *Acinetobacter baumannii*. *Prais-Winstein Regression (p < 0.05).
