# Supplementary material for: Use of monitoring indicators in hospital management of antimicrobials
Source: BMC Infect Dis. 2021 Aug 17;21:827. doi: 10.1186/s12879-021-06542-5 (PMC8369325; doi:10.1186/s12879-021-06542-5)
Supplement: Supplementary file 3 — Additional file 3. Global distribution of diagnoses related to the use of antimicrobials, 2018. [file 12879_2021_6542_MOESM3_ESM.docx]

**Additional file 3.** Global distribution of diagnoses related to the use of antimicrobials, 2018.

| **DIAGNOSTIC** | **%** |
| --- | --- |
| Surgical prophylactic | 35.7 |
| Respiratory infection | 24.1 |
| Sepsis | 14.3 |
| Skin/soft tissue infection | 7.6 |
| Urinary infection | 7.1 |
| Abdominal infection | 5.9 |
| Others | 5.3 |

**Legend:** Others - surgical site infection, visceral leishmaniasis, febrile neutropenia, bacterial endocarditis.
