# Supplementary material for: Use of monitoring indicators in hospital management of antimicrobials
Source: BMC Infect Dis. 2021 Aug 17;21:827. doi: 10.1186/s12879-021-06542-5 (PMC8369325; doi:10.1186/s12879-021-06542-5)
Supplement: Supplementary file 4 — Additional file 4. Temporal trends in the use of antimicrobials, by hospital ward, 2018. [file 12879_2021_6542_MOESM4_ESM.docx]

**Additional file 4.** Temporal trends in the use of antimicrobials, by hospital ward, 2018.

| **VARIABLES** | **WARDS** | | | | | | | | | |
| --- | --- | --- | --- | --- | --- | --- | --- | --- | --- | --- |
|  | **SUR** | | **MED** | | **PNE/INF** | | **PED** | | **ICU** | |
|  | **Monthly Variation** | **p-value** | **Monthly Variation** | **p-value** | **Monthly Variation** | **p-value** | **Monthly Variation** | **p-value** | **Monthly Variation** | **p-value** |
| **Use of antimicrobials (%)** | -1.6 | 0.005* | 2.6 | 0.021* | 0.4 | 0.746 | -2.6 | 0.211 | 0.4 | 0.660 |
| **Number of prescriptions (%)** |  |  |  |  |  |  |  |  |  |  |
| First-generation cephalosporins IV/PO | -1.5 | 0.133 | 0.2 | 0.620 | -0.3 | 0.008* | 0.8 | 0.326 | 0.8 | 0.301 |
| Fluoroquinolone IV/PO | -0.0 | 0.966 | 0.6 | 0.255 | 0.5 | 0.437 | 0.1 | 0.834 | -0.6 | 0.278 |
| Carbapenems IV | 0.4 | 0.080 | 1.0 | 0.002* | -0.2 | 0.494 | 0.0 | 0.898 | 0.8 | 0.142 |
| Third-generation cephalosporins IV | 0.5 | 0.221 | -0.6 | 0.393 | -0.1 | 0.790 | -0.1 | 0.519 | -0.5 | 0.324 |
| Metronidazole IV/PO | 0.9 | 0.181 | 0.4 | 0.425 | 0.9 | 0.027* | -0.5 | 0.208 | 0.3 | 0.605 |
| Cefepime IV | 0.2 | 0.272 | 0.5 | 0.544 | -0.2 | 0.786 | -0.1 | 0.665 | -0.0 | 0.990 |
| Clindamycin IV | -0.3 | 0.737 | -0.1 | 0.805 | -0.0 | 0.951 | -0.4 | 0.074 | -1.4 | 0.139 |
| Antifungals IV/PO | -0.1 | 0.113 | -0.0 | 0.937 | 0.5 | 0.567 | -0.3 | 0.298 | 0.1 | 0.693 |
| Glycopeptides IV | 0.4 | 0.050 | 0.2 | 0.738 | -0.2 | 0.254 | -0.2 | 0.428 | 0.2 | 0.688 |
| Azithromycin IV/PO | - | - | -0.4 | 0.275 | -0.2 | 0.772 | 0.9 | 0.004* | 0.2 | 0.402 |
| Penicillins^1^ IV/PO | -0.0 | 0.879 | -1.9 | 0.001* | 0.7 | 0.374 | 0.5 | 0.465 | 0.0 | 0.940 |
| Co-trimoxazole IV/PO | - | - | -0.5 | 0.067 | 0.3 | 0.457 | -0.0 | 0.911 | 0.1 | 0.562 |
| Aminoglycosides IV | -0.1 | 0.037* | 0.4 | 0.079 | -1.1 | 0.017* | -0.4 | 0.309 | -0.5 | 0.167 |
| **ID DOT/1000pd** |  |  |  |  |  |  |  |  |  |  |
| Fluoroquinolone IV/PO | -1.2 | 0.788 | -0.7 | 0.858 | 2.7 | 0.493 | -2.3 | 0.327 | -14.5 | 0.138 |
| First-generation cephalosporins IV/PO | -10.6 | 0.000* | 0.7 | 0.077 | -0.6 | 0.490 | 4.2 | 0.133 | 0.7 | 0.875 |
| Carbapenems IV | 1.2 | 0.122 | 5.6 | 0.009* | -8.8 | 0.065 | 3.4 | 0.153 | 24.4 | 0.044* |
| Third-generation cephalosporins IV | 2.6 | 0.402 | 0.3 | 0.914 | -2.8 | 0.507 | 0.6 | 0.876 | -6.3 | 0.161 |
| Cefepime IV | 0.2 | 0.864 | 4.2 | 0.116 | -4.4 | 0.550 | -3.5 | 0.323 | -2.1 | 0.782 |
| Metronidazole IV/PO | 2.1 | 0.642 | 1.0 | 0.454 | 0.7 | 0.546 | 0.6 | 0.882 | 5.6 | 0.394 |
| Antifungals IV/PO | -0.4 | 0.113 | 0.7 | 0.682 | 5.1 | 0.244 | -1.8 | 0.783 | 1.7 | 0.873 |
| Co-trimoxazole IV/PO | - | - | -0.2 | 0.693 | 10.7 | 0.166 | -2.4 | 0.433 | 3.7 | 0.572 |
| Clindamycin IV | -2.1 | 0.700 | 0.4 | 0.884 | -0.7 | 0.831 | -3.1 | 0.072 | -23.5 | 0.015* |
| Glycopeptides IV | 1.7 | 0.046* | 0.5 | 0.882 | -0.3 | 0.899 | -0.1 | 0.914 | 4.3 | 0.738 |
| Aminoglycosides IV | -1.8 | 0.037* | 0.5 | 0.829 | -7.3 | 0.008* | -2.1 | 0.443 | -12.8 | 0.157 |
| Penicillins^1^ IV/PO | -0.0 | 0.879 | -4.9 | 0.034* | 2.1 | 0.577 | 2.9 | 0.383 | 0.0 | 0.996 |
| Azithromycin IV/PO | - | - | -0.5 | 0.693 | -1.3 | 0.727 | 3.6 | 0.021* | 3.2 | 0.272 |
| **ID LOT/1000pd** | -7.1 | 0.159 | 1.3 | 0.717 | -3.8 | 0.723 | 3.5 | 0.599 | -5.1 | 0.637 |
| **ID DOT/LOT ratio** | 0.0 | 0.515 | 0.0 | 0.165 | -0.0 | 0.607 | -0.0 | 0.448 | -0.0 | 0.980 |
| **Antimicrobial resistance** |  |  |  |  |  |  |  |  |  |  |
| Methicillin ^R^ | 0.1 | 0.002* | 0.0 | 0.547 | 0.2 | 0.175 | 0.0 | 0.878 | 1.0 | 0.024* |
| Carbapenem ^R^ | - | - | -0.3 | 0.058 | - | - | 0.0 | 0.636 | 0.3 | 0.432 |

**Legend:** ID – Incidence Density, SUR - Surgical Clinic, MED - Medical Clinic, PNE/INF - Pneumology / Infectology, PED - Pediatrics, ICU - Intensive Care Unit. *Prais-Winstein Regression (p < 0.05). ^1^ penicillin/penicillin with beta lactamase inhibitor.
